# Porcelain Contours

**Published:** 1894-05

**Authors:** George F. Grant

**Affiliations:** Boston


### PORCELAIN CONTOURS.[1]

BY GEORGE F. GRANT, D.M.D., BOSTON.

THE ideas to which I wish to call your attention this evening have been taking form in my mind for some years past.

It has been my thought for some time that we should be able to more nearly approach natural appearance in the restoration of those portions of the anterior teeth which have been lost through decay or fracture. We have, perhaps, been too conservative in this matter in aiming at permanence to the sacrifice of æsthetic considerations.

Doubtless you will argue to this proposition that no contour gold filling, however skilfully made, has anything but its technical perfection to recommend it, and is therefore a failure because it simply replaces one defect in appearance with another.

The great need of our profession to-day is, I think, a new filling-material. The properties it should possess are too well understood to need consideration here. That we shall have it before many years is a safe prophecy.

While many methods of restoring the labial surfaces of the anterior teeth with porcelain inlays have been and are employed, we cannot say that our success has been satisfactory to our best feeling. The subject of inlays may be left, however, for future consideration.

---

[1] Read before the Harvard Odontological Society, December 28, 1893.

Many cases have occurred in my practice where gold could not be used to restore the lost portions of front teeth. Sometimes the necessary retaining cavities could not be made; in other cases patients have objected to the use of gold on account of the unsightly appearance of such large contours made of it.

My purpose in offering this paper is to bring about a comparison of views as to the best method of procedure in such cases. My efforts in this line have been attended with a widely-varied success.

For an example we will take a central incisor which has been broken; say one-third is missing either diagonally from approximal surface to cutting-edge, or that it is broken in a line parallel with the cutting-edge. It is very desirable to restore such a tooth with the least possible disfigurement, also, if possible, without devitalizing the pulp. The first step is to make the base or fractured surface as nearly a plane as is possible. This insures the maximum of strength in the porcelain tip which is to be used in contouring. Wherever possible, it is desirable to obtain sufficient width labio-lingually to allow of the insertion of at least two pins or dowels, which I place at points quite near the approximal surfaces in a transverse fracture, being governed in other cases by existing conditions. For instance, in case of a fracture which took a diagonal direction, such as to leave a weak corner on the tip, I should not hesitate to sacrifice a little of the natural tooth by cutting back vertically, so as to reduce the obliqueness. By this course much strength of body in the tip is gained, as well as the advantage of being able to insert both dowels in a line with the axis of the tooth. This cutting of the tooth is much less painful if performed with fine corundum wheels.

I have lately been experimenting with wheels made of a metal charged with diamond-dust. The advantage they seem to possess over any other instrument is that they are much more rapid in operation, give less vibration, and cut a perfectly accurate angle or curve, which cannot be obtained with any other wheel. I wish to say, incidentally, here, that no form of chisel or coarse wheel can be expected to make a perfect or even a good surface on a hard surface like enamel.

Until I began using corundum wheels for shaping the edges of cavities to receive gold fillings there was a feeling of insecurity in my mind as to what kind of a result might be expected.

In using instruments charged with diamond-dust I prefer water to any other medium on the natural teeth, using oil only in drilling porcelain.

Having prepared the surface carefully, the next step is drilling pits for the dowels. The drill used should be slightly longer than the wire of which the dowels are made. If a twist drill—which is the best form—is used, it should be protected by a guard set at the desired depth to prevent going too deeply. I use carbolic acid, full strength, on a drill for the natural tooth in the same manner as on the diamond drill in porcelain. It seems to allay the pain quite considerably.

After drilling, the dowels are placed in position and an impression taken in which the dowels are withdrawn. To the cast made from this impression the porcelain tip or contour is accurately fitted. It may be carved and fired or it may be made by cutting a piece from a porcelain tooth of the exact shade desired. It is so much easier to cut from a tooth than to carve the contour, that I always adopt that plan. Porcelain is easily cut with rubber and corundum disks if plenty of water is used with them. True, they can be cut more accurately with the diamond disk, but until lately diamond tools were too uncertain and expensive. The firm of Godber & Parker, of Waltham, have of late been making some very fine work for me in this line at a very moderate cost. I have been able to get from them just such instruments as I have always wished for but never before obtained.

We have now the cast, with the pins in position, also one bit of porcelain, which is to be fitted to it. First withdraw the pins from the cast, then fit by grinding until contact is as perfect as can be obtained. The points at which the holes are to be drilled in the porcelain are then marked, and when drilled the parts should come together with the platina dowels in position.

There are two ways of procedure from this point. The first is to simply set the dowels in the tooth with zinc phosphates, and afterwards set the tip upon them with the same material.

This is not so good, however, as to fuse the pins into the porcelain with some powdered glass or other material like the Downie crown flux, which melts at quite a low temperature. The strength of the porcelain tip is thus greatly increased, and there is another quite important point gained,—that the tip so treated is more transparent than one in which the pins are set with phosphate. While the labial surfaces of the tip should not present any prominence, it is quite possible that the lingual surface might overhang slightly; in fact, it would be better to leave it so until the point was firmly fixed in the tooth and finish it down the last thing with fine corundum, either wheels or wood points charged with corundum powder.

Another form of contour is where a considerable portion of the tooth has been lost through caries of the approximal surface. The cases present some features which require a little different treatment. They are prepared almost in the same manner as for a gold contour, except that, if possible, the edges are made perfectly flat. Usually there is no opportunity for the drilling of dowel-holes. This difficulty is met in the following manner: After the porcelain contour is accurately fitted, a deep slot is cut nearly the whole length of basal surface. Into this slot is inserted a strip of platina doubled or folded together so that the edges projecting from the slot open like the leaves of a book. When this platina is fused into the porcelain the edges can be turned back at any angle, this giving a firm support and means of secure attachment to the tooth.

I have also found a slight modification of this idea quite serviceable in labial inlays where the cavity was of considerable depth.

In any case where the drilling of a porcelain piece may be attended with danger, a slot can be safely cut and a flattened pin or other bit of platina as securely fastened by baking as though inserted in a drilled cavity.

Operations of this kind are very satisfactory to the eye, and possess considerable strength. It is not claimed that they are as strong as gold contours can be made, but it is an open question whether they do not on the whole compare favorably when all things are considered. They relieve both patient and operator of the most disagreeable features incident to protracted sittings, as the greater part of the work is done out of the mouth.

As to the instruments which I have shown, I will say that some of them are first attempts of the makers, and will be greatly improved in the future. The diamond saws are made after the style of the Merriam saw. Even in their present imperfect state they are an immense improvement over any other instrument which I have seen. They run smoothly, having none of that extremely unpleasant rasp inseparable from a toothed wheel. When kept wet with water or carbolic acid and alcohol they produce very little pain, even in amputation of a live tooth. In fact, a little close observation seems to establish the feeling that a large part of the pain incident to dental operations is so clearly the result of nervous irritation, induced by the noise and vibration of coarse-cutting engine-tools, that an improvement in this direction is very much to be desired.

